# Hospital-Based Seroprevalence and Socio-Environmental Predictors of Human Leishmaniasis in Baringo County, Kenya: Implications for One Health Surveillance and Control

**DOI:** 10.3390/pathogens15101026

**Published:** 2026-09-30

**Authors:** Hellen Njeri Maingi, James Ng’ang’a Chege, Richard B. Yapi, Davis Njuguna Karanja, Maingi Ndichu, Helena Ngowi, Damaris Matoke-Muhia, Bassirou Bonfoh

**Affiliations:** 1 Department of Veterinary Pathology, Microbiology and Parasitology, Faculty of Veterinary Medicine, University of Nairobi, Nairobi P.O. Box 29053-00625, Kenya; jchege@uonbi.ac.ke (J.N.C.); dnkaranja@uonbi.ac.ke (D.N.K.); nmaingi@uonbi.ac.ke (M.N.); 2 Centre d’Entomologie Médicale et Vétérinaire, Université Alassane Ouattara, Bouaké P.O. Box 01 BP 1303, Côte d’Ivoire; richard.yapi@csrs.ci; 3 Department of Veterinary Medicine and Public Health, Sokoine University of Agriculture, Morogoro P.O. Box 3000, Tanzania; helenangowi@gmail.com; 4 Center for Biotechnology Research and Development, Kenya Medical Research Institute, Nairobi P.O. Box 54840-00200, Kenya; dmatoke@kemri.go.ke; 5 Centre Suisse de Recherches Scientifiques en Côte d’Ivoire (CSRS), Abidjan P.O. Box 01 BP 1303, Côte d’Ivoire; bassirou.bonfoh@csrs.ci

**Keywords:** human visceral leishmaniasis, *Leishmania* seroprevalence, RDT, ELISA, socio-environmental risk factors, Baringo County, Kenya, epidemiological surveillance, One Health

## Abstract

Background: Leishmaniasis is a vector-borne neglected tropical disease caused by obligate intracellular protozoan parasites of the genus *Leishmania* and transmitted by bites from infected female phlebotomine sand flies. Visceral leishmaniasis (VL), the most severe clinical form, remains a major public health concern in Eastern Africa and a priority for control and elimination. Although Baringo County is a well-established endemic focus in Kenya, the spatial distribution of human exposure and the socio-environmental determinants of infection remain inadequately characterized. This hospital-based cross-sectional study therefore estimated *Leishmania* seroprevalence. It assessed the sociodemographic, behavioral, and environmental predictors of seropositivity among patients presenting with clinical features suggestive of VL in two endemic sub-counties of Baringo County. Methods: A hospital-based cross-sectional study was conducted from January to March 2026 at Marigat and Chemolingot Sub-County Hospitals. We enrolled 260 patients with clinical features suggestive of VL through systematic sampling and tested them using the rK39 rapid diagnostic test (RDT) and an indirect enzyme-linked immunosorbent assay (ELISA). We collected sociodemographic, behavioral, household, and environmental data using a structured questionnaire. Associations between explanatory variables and ELISA seropositivity were evaluated using Pearson’s chi-square test or Fisher’s exact test. We included variables with *p* < 0.25 in multivariable logistic regression to identify independent predictors of seropositivity. Agreement between the rK39 RDT and ELISA was assessed using Cohen’s kappa statistics. We confirmed classical positive cases using bone marrow aspirates. Results: Overall *Leishmania* seroprevalence was 18.5% (95% CI: 14.2–23.7) by rK39 RDT and 20.8% (95% CI: 16.2–26.2) by ELISA. ELISA seroprevalence was substantially higher in Tiaty Sub-County (36.6%) than in Baringo South Sub-County (4.7%). Residence in Tiaty was independently associated with markedly higher odds of seropositivity than residence in Baringo South [aOR] = 11.30, 95% CI: 1.96–64.97; *p* = 0.007. Compared with children aged < 15 years, participants aged 15–44 years had lower odds of seropositivity (aOR = 0.30, 95% CI: 0.11–0.93; *p* = 0.037). Residents without a dog had higher odds of seropositivity (aOR = 3.17, 95% CI: 1.06–9.50; *p* = 0.039) than those with a dog. After adjustment, no other predicted factors were independently associated with seropositivity, suggesting that geographical differences may have confounded the unadjusted associations. Conclusion: *Leishmania* seroprevalence was substantial and unevenly distributed, with Tiaty Sub-County and children under 15 years bearing the greatest burden. Therefore, targeted, child-focused surveillance and integrated One Health interventions, including early case detection, improved housing, personal protection, vector-habitat management, and community education, are needed to strengthen VL control and advance the WHO 2030 targets for the Neglected Tropical Diseases Roadmap 2021–2030.

## 1. Introduction

Leishmaniasis is a vector-borne neglected tropical disease caused by obligate intracellular protozoan parasites of the genus *Leishmania*. More than 20 *Leishmania* species cause human disease and are transmitted by over 90 species of phlebotomine sand flies, principally *Phlebotomus* in the Old World and *Lutzomyia* in the New World [1,2,3]. Environmental change, urbanization, population movement, and climate change can alter vector distribution and seasonality, promoting disease emergence and re-emergence. Leishmaniasis disproportionately affects underserved populations, among whom poverty, malnutrition, poor housing, weak health systems, and limited access to healthcare intensify its clinical and socioeconomic burden [4].

The *Leishmania* parasite is transmitted through complex anthroponotic or zoonotic cycles involving phlebotomine sand fly vectors, mammalian hosts, and environmental conditions favorable to vector survival. During a blood meal, an infected female sand fly inoculates metacyclic promastigotes into the host’s skin [5]. After inoculation, macrophages engulf promastigotes, which transform into intracellular amastigotes that multiply and may disseminate to the spleen, liver, bone marrow, and lymph nodes [6]. Sand flies occupy sylvatic, domestic, and peri-domestic environments and obtain blood meals from humans and other vertebrate hosts, including domestic animals [3,7]. Parasite species, host immunity, nutritional status, comorbidities, and environmental exposure influence infection outcome [8]. 

Leishmaniasis occurs mainly in cutaneous, mucocutaneous, and visceral forms, while post-kala-azar dermal leishmaniasis may develop after visceral leishmaniasis (VL). VL, the most severe manifestation, is caused mainly by the *L. donovani* complex that predominates in Eastern Africa and the Indian subcontinent, whereas *L. infantum* occurs mainly in the Mediterranean region and Latin America [9]. Clinical manifestations range from asymptomatic infection to prolonged irregular fever, weight loss, pallor, hepatosplenomegaly, and pancytopenia. Untreated symptomatic VL is fatal in more than 95% of cases, while HIV coinfection increases the risks of severe disease, treatment failure, relapse, and mortality [10,11]. 

VL is maintained through two principal epidemiological cycles. Anthropo-zoonotic VL is caused predominantly by *L. donovani*, with humans serving as the main reservoir of infection. In contrast, zoonotic VL is caused primarily by *L. infantum*, with domestic dogs as the principal reservoir host [12]. Emerging evidence that dogs and other mammals may influence transmission in some endemic settings in Kenya highlights the need to investigate potential animal reservoirs within a One Health framework [5,13].

Despite leishmaniasis being preventable and treatable, it remains a major global public health challenge, particularly in Eastern Africa, the Indian subcontinent, and Brazil. The disease is endemic in nearly 100 countries, with more than 350 million people at risk and an estimated 12 million infections worldwide [14,15]. Ethiopia, Kenya, Somalia, South Sudan, Sudan, and Uganda are major endemic countries and collectively account for a substantial proportion of reported VL cases in Eastern Africa [16]. However, the true regional burden is likely underestimated because of inadequate surveillance, limited diagnostic capacity, incomplete case reporting, and substantial clinical and epidemiological heterogeneity [11,17].

In Kenya, approximately six million people are at risk of VL, particularly in arid and semi-arid endemic counties. Baringo County is a long-established focus where *Phlebotomus martini* principally transmits *L. donovani*. Its semi-arid ecology—including termite mounds, cracked soils, animal burrows, and *Acacia–Balanites* vegetation—provides suitable sand fly breeding and resting habitats [18,19,20]. These ecological conditions interact with pastoral livelihoods, outdoor sleeping, traditional housing, proximity to livestock shelters, inadequate vector-control coverage, low educational attainment, and malnutrition, potentially increasing human–vector contact and susceptibility to infection [13,20].

Effective surveillance and diagnosis are complicated by the clinical similarity of VL to malaria, tuberculosis, typhoid fever, and other febrile illnesses [10,15,21,22]. Parasitological confirmation relies on the microscopic detection of *Leishmania* amastigotes in splenic, bone marrow, or lymph-node aspirates. Although splenic aspiration is highly sensitive, it requires specialized expertise and carries a risk of internal bleeding. Bone marrow aspiration is safer but painful and less sensitive, whereas lymph-node aspiration is less invasive but may miss early or mild infections [23,24]. Molecular methods offer high sensitivity and species identification, but cost, infrastructure, and technical requirements limit their routine use in resource-constrained settings [25,26].

Serological methods therefore provide practical diagnostic alternatives in resource-limited endemic settings. The recombinant kinesin antigen-39 rapid diagnostic test (rK39 RDT) enables rapid point-of-care testing, whereas enzyme-linked immunosorbent assay (ELISA) provides standardized laboratory detection of anti-*Leishmania* antibodies [20,27,28,29]. However, performance varies geographically because of parasite genetic diversity, antigen composition, and differences in host antibody responses [9]. Moreover, antibodies may persist after treatment; therefore, serological tests cannot reliably distinguish active disease from previous exposure or asymptomatic infection [30]. Serological findings must therefore be interpreted alongside clinical presentation, treatment history, and relevant differential diagnoses.

Despite persistent VL transmission in Baringo County, the magnitude, geographical distribution, and local determinants of human *Leishmania* seropositivity remain inadequately characterized. This hospital-based cross-sectional study estimated seroprevalence using the rK39 RDT and ELISA. It assessed the sociodemographic, behavioral, household, and environmental predictors of seropositivity among patients with clinical features suggestive of VL. The findings aimed to identify high-risk populations and transmission hotspots and inform geographically targeted surveillance and integrated One Health control strategies in Baringo County, Kenya. 

## 2. Materials and Methods

### 2.1. Study Area

The study was conducted in Baringo South and Tiaty sub-counties in Baringo County, Kenya, where recurrent outbreaks of VL have been documented for more than three decades, an established endemic focus for both visceral and cutaneous leishmaniasis [15,20,31]. Baringo County lies within Kenya’s Great Rift Valley and is characterized by a semi-arid climate, extensive *Acacia–Balanites* woodlands, abundant termite mounds, animal burrows, and rock crevices. These ecological features provide favorable breeding and resting habitats for *Phlebotomus* sand flies. Traditional mud-walled and grass-thatched houses, particularly those with cracked walls, also provide indoor resting sites for sand flies and may increase human exposure to infective bites [13,19,32]. 

Pastoralism is the predominant livelihood in the study area. Seasonal movements of households and livestock may increase exposure to sand fly habitats and limit consistent access to preventive interventions, disease surveillance, and healthcare services [20,33,34]. Livestock grazing, sleeping outdoors during hot nights, resting beneath Acacia trees, and keeping livestock and dogs near human dwellings may further increase exposure to sand flies. *Acacia* trees may support vector survival by providing sugar sources, moisture, and suitable resting microhabitats, thereby increasing sand fly abundance around household compounds [11]. Domestic dogs have also been investigated as potential reservoir hosts in East Africa, particularly in settings with limited veterinary services and vector control measures [27].

**Figure 1:**
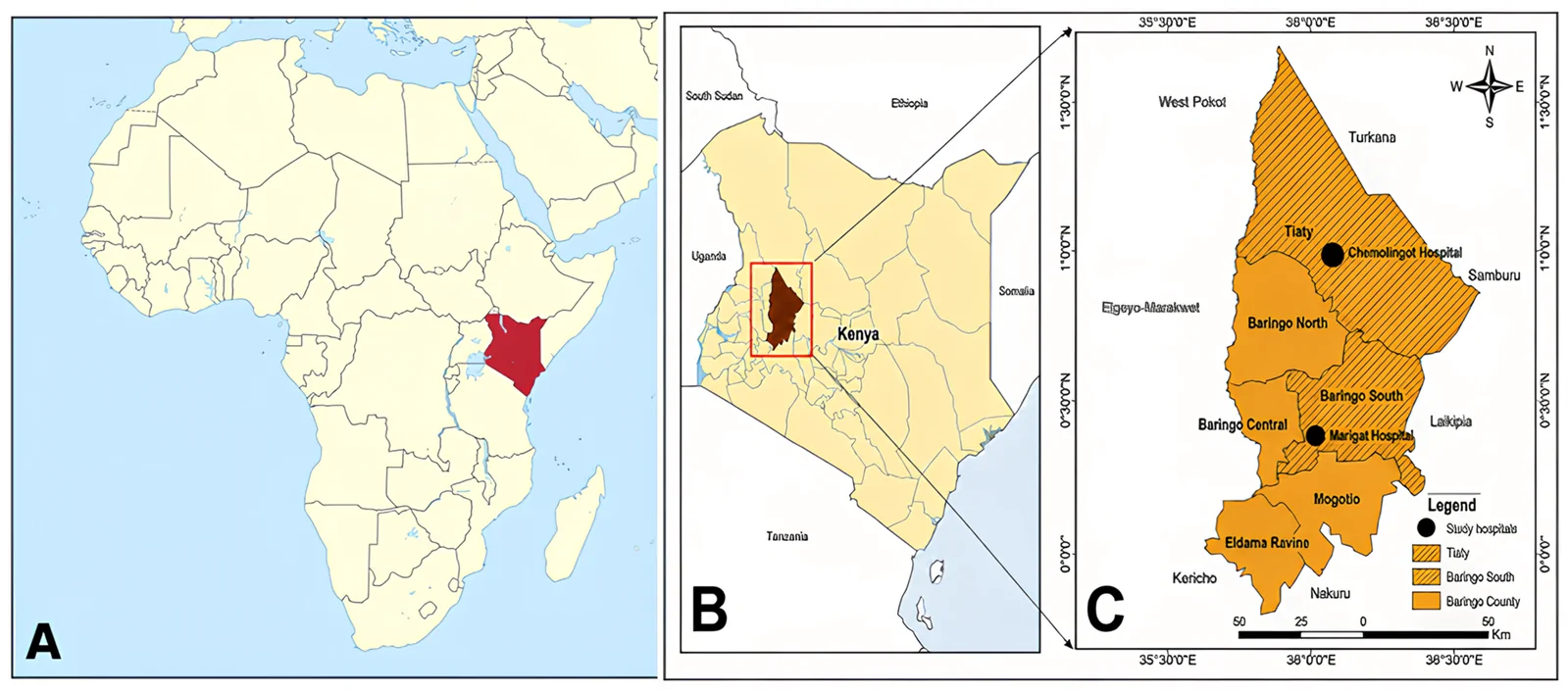
Map of Africa (**A**) showing Kenya and inset (**B**) the map of Kenya showing the location of Baringo County and inset (**C**) showing the study location, its sub-counties, and the neighboring counties.

The county borders Turkana, West Pokot, Samburu, Laikipia, Nakuru, Kericho, Uasin Gishu, and Elgeyo-Marakwet counties and is administratively divided into seven sub-counties (Figure 1).

### 2.2. Study Design

A hospital-based cross-sectional sero-epidemiological study was conducted from January 2025 to March 2026 among patients presenting clinical features suggestive of VL. This design was selected because it enabled the estimation of *Leishmania* seroprevalence and the assessment of factors associated with seropositivity within a defined study period [20].

#### 2.2.1. Justification for the Hospital-Based Study Design

The study recruited patients with VL-compatible symptoms from Marigat Sub-County Hospital in Baringo South and Chemolingot Sub-County Hospital in Tiaty. These principal referral facilities serve predominantly pastoral populations in endemic areas and enabled standardized participant selection, clinical assessment, blood collection, evaluation of relevant differential diagnoses, confirmatory diagnosis by parasitology, evaluation of potential risk factors under controlled conditions, and *Leishmania*-specific testing [10,15,20]. Hospital-based recruitment was appropriate given the population’s geographic dispersion and pastoral population for whom community-based surveys may be constrained by difficult terrain, mobility, and limited accessibility [27,35].

#### 2.2.2. Participant Recruitment and Sampling Strategy

A multidisciplinary team comprising a study clinician, laboratory technologist, and two trained research assistants recruited participants and collected data at the two study hospitals. The team identified potential participants through routine clinical assessment and review of outpatient registers. We defined a suspected VL case as irregular fever lasting at least two weeks, accompanied by at least one compatible clinical feature, including weight loss, splenomegaly, hepatomegaly, lymphadenopathy, anemia, anorexia or poor appetite, vomiting, epistaxis, or headache.

We selected participants using systematic random sampling: we chose a random starting point at the beginning of each clinic day, then invited every third eligible patient to participate. We escorted eligible patients to a private consultation room, explained the study procedures, and obtained written informed consent before enrolment.

#### 2.2.3. Diagnostic Algorithm for Visceral Leishmaniasis Applied in This Study

Patients meeting the suspected VL case definition were initially tested for malaria using an RDT and/or blood-film microscopy. We treated malaria-positive patients appropriately and reassessed them if fever or splenomegaly persisted, while malaria-negative patients proceeded to rK39 testing. Other differential diagnoses, including tuberculosis, typhoid fever, and brucellosis, were considered because *Leishmania* seropositivity alone cannot distinguish active disease from previous or asymptomatic infection. We conducted parallel ELISA testing for study purposes, and the two serological assays showed strong agreement. A positive rK39 result supported VL diagnosis in previously untreated patients with compatible clinical features. Patients with negative serological results but persistent clinical suspicion underwent bone marrow aspiration for parasitological confirmation. Bone marrow examination was also performed in cases of suspected relapses or treatment failure because antibodies may remain detectable after successful treatment.

### 2.3. Inclusion Criteria and Exclusion Criteria

Eligible participants were residents of Baringo South or Tiaty sub-counties for at least 6 months before enrollment. Patients of either sex and any age presenting to the outpatient department provided written informed consent (or parental consent with child assent for minors). They agreed to provide a venous blood sample. Individuals who had received anti-leishmanial treatment within the preceding three months, required emergency medical care before recruitment, or were non-residents of the study area were excluded.

### 2.4. Sample Size Determination

The minimum sample size was determined using the formula for estimating prevalence in a cross-sectional study as described by Fosgate, based on a similar geographical location and socio-economic set-up [35] and a seroprevalence estimate of 21% from a similar study in West Armachiho District, Northwest Ethiopia [36].

(1) $$n=\frac{Z^{2}pq}{e^{2}}$$

where Z = 1.96 for a 95% confidence level, *p* = 0.208 (the expected seroprevalence of visceral leishmaniasis based on previous studies in Baringo County), q = 1 − *p*, and e = 0.05 (the desired margin of error). The minimum sample size was 255 participants. However, the study enrolled 260 participants, exceeding the minimum required sample size and providing adequate precision for estimating the seroprevalence of *Leishmania* infection and sufficient observations for subsequent risk-factor analyses.

### 2.5. Informed Consent and Ethical Considerations

The study team explained the procedures, risks, and benefits to participants in their preferred language. The researchers obtained written informed consent from adults and parental or guardian consent and age-appropriate assent for participants younger than 18 years. Participation was voluntary, withdrawal did not affect access to medical care, and all participant information was anonymized and kept confidential.

Ethical approval was granted by the Kenyatta National Hospital–University of Nairobi Ethics and Research Committee (Protocol No. P117/02/2025), with research authorization from the National Commission for Science, Technology and Innovation (License No. NACOSTI/P/25/4175423). The researchers obtained administrative approval from the Baringo County Department of Health, the participating hospitals, and relevant local authorities.

### 2.6. Data Collection

Data were collected using a structured, interviewer-administered questionnaire and a review of relevant hospital records. The questionnaire was developed in English, translated into Kiswahili, Kalenjin, and back-translated to ensure semantic equivalence. We pretested it among 20 patients excluded from the final analysis and made minor revisions to improve clarity and consistency.

The questionnaire captured participants’ sociodemographic characteristics, including age, sex, education, occupation, place of residence, and comorbidities; household characteristics, including wall, roofing, and flooring materials and wall condition; environmental exposures, such as proximity to termite mounds, *Acacia* vegetation, and domestic animals; and behavioral factors, including outdoor sleeping, evening outdoor activities, mosquito-net use, and sleeping near domestic animals. Clinical information obtained from the questionnaire and hospital records included presenting features consistent with VL, diagnostic findings, treatment received, referral status, and clinical outcomes.

Attending clinicians assessed all suspected cases according to the study case definition and routine hospital diagnostic procedures. Participants were allocated proportionately between the two hospitals based on their average outpatient caseloads, and recruitment continued until the predetermined sample size was attained.

### 2.7. Sample Collection and Processing

#### 2.7.1. Blood Sample Collection

Trained phlebotomists aseptically collected approximately 5 mL of venous blood from each participant and placed it in EDTA vacutainer tubes. We centrifuged the blood samples at 3000 rpm for 10 min to separate plasma, aliquoted it into appropriately labeled cryovials, and stored it at −20 °C. We then triple-packaged whole-blood and plasma aliquots and transported them in a cooler box under cold-chain conditions to the Kenya Medical Research Institute (KEMRI) in Nairobi for polymerase chain reaction and enzyme-linked immunosorbent assay analyses.

All serum samples were initially screened using the ACRO Biotech *Leishmania* Rapid Test, an rK39-based lateral-flow immunochromatographic assay for the qualitative detection of anti-*Leishmania* IgG and IgM antibodies, following the manufacturer’s instructions. The assay has a reported sensitivity of 92.05% and specificity of approximately 98%. Samples that tested positive were stored at −80 °C pending confirmatory ELISA testing.

Complementary serological testing was performed using a commercial indirect ELISA kit (LEISHMANIA ELISA IgG+IgM; Vircell S.L., Granada, Spain) to detect anti-L: *infantum* IgG and IgM antibodies. Samples and kit controls were tested in duplicate according to the manufacturer’s instructions, and optical density was measured at 450 nm. Antibody indices were classified as negative (<9), equivocal (9–11), or positive (>11), with equivocal samples retested. ELISA positivity was interpreted as evidence of anti-*Leishmania* antibodies, not definitive confirmation of active VL.

Although the direct agglutination test (DAT) is a recognized field diagnostic for visceral leishmaniasis and has been included in the WHO Model List of Essential In Vitro Diagnostics since 2021 [23,37], ELISA was selected as a standardized, objective, and operationally feasible alternative [29]. DAT requires serial dilutions, prolonged incubation, and access to quality-controlled antigen. Although the ELISA used *L. infantum* promastigote antigens, it can detect antibodies against *L. donovani*, the principal cause of VL in Kenya, because both species belong to the *L. donovani* complex and share antigenic determinants. Accordingly, *L. infantum*-derived antigens have been successfully used for VL serodiagnosis in East Africa [9,29]. ELISA-based assays have also demonstrated high sensitivity (93–100%) and specificity (97–98%) in endemic settings. Although the kit has not been formally validated in Kenya, its strong agreement with rK39 in this study (95.4%; κ = 0.854) supports the internal consistency of the findings. Nevertheless, future studies should validate its performance against a parasitological or molecular reference standard.

#### 2.7.2. Bone Marrow Aspiration

Bone marrow aspirates were collected aseptically by a trained clinician from patients presenting classical clinical features of visceral leishmaniasis and those with suspected relapses. Following disinfection of the posterior iliac crest and administration of local anesthesia, a sterile aspiration needle was advanced into the marrow cavity, and a small volume of bone marrow was aspirated into a sterile syringe. Thin smears were prepared immediately to prevent clotting, air-dried, fixed, and stained with Giemsa or *Leishman* stain. The slides were examined under oil-immersion microscopy, and parasitological confirmation was based on the detection of intracellular or extracellular *Leishmania* amastigotes (*Leishman–Donovan* bodies), identified by their characteristic nucleus and rod-shaped kinetoplast [25]. Bone marrow aspiration was the preferred routine method for parasitological confirmation because it was safer and more feasible in participating sub-county hospitals. Trained clinicians performed the procedure under local anesthesia and aseptic conditions. Although splenic aspirate microscopy has greater diagnostic sensitivity, it carries a risk of potentially fatal hemorrhage and requires specialized expertise, blood-transfusion capacity, surgical support, and intensive post-procedure monitoring [15]. Trained microscopists systematically examined the bone marrow smears to optimize diagnostic sensitivity.

### 2.8. Data Management and Statistical Analysis

Data obtained from interviewer-administered structured questionnaires and laboratory assays, the rK39 immunochromatographic rapid diagnostic test (RDT), and enzyme-linked immunosorbent assay (ELISA) were independently double-entered into Microsoft Excel 2019. We compared, reconciled, coded, de-identified, and checked the datasets for completeness, consistency, outliers, and missing values, and analyzed them using Stata version 14 (StataCorp LLC, College Station, TX, USA).

We summarized participant characteristics using frequencies and percentages for categorical variables and means with standard deviations or medians with interquartile ranges for continuous variables, as appropriate. *Leishmania* seroprevalence was estimated separately for the rK39 RDT and ELISA and reported with 95% confidence intervals (CIs), with ELISA seropositivity serving as the primary outcome for risk-factor analysis [38,39].

Associations between ELISA seropositivity and potential risk factors, including study site, age, sex, educational attainment, occupation, housing type, sleeping practices, domestic animal ownership, and ecological habitat, were assessed using Pearson’s chi-square or Fisher’s exact test and univariable logistic regression [40].

Variables with *p* ≤ 0.25 in bivariable analysis, together with factors considered epidemiologically relevant, were included in the multivariable logistic regression model. This inclusive threshold, consistent with the purposeful-selection approach, reduced the risk of excluding potentially important predictors or confounders whose effects might become apparent after adjustment. Multicollinearity was assessed before model fitting. We reported crude and adjusted odds ratios with 95% confidence intervals and defined statistical significance in the final model as a two-sided *p* < 0.05. We evaluated model fit and predictive performance using the likelihood-ratio test, Hosmer–Lemeshow goodness-of-fit test, pseudo-R^2^, and classification measures [41,42]. We removed variables that did not remain statistically significant after adjustment and assessed model fit and discrimination (model AUC = 0.87).

We assessed agreement between the rK39 RDT and ELISA using Cohen’s kappa and observed percentage agreement. We calculated the sensitivity, specificity, positive predictive value, and negative predictive value of the rK39 RDT using ELISA as the comparator. Because ELISA is not a definitive gold standard for active VL, these estimates were interpreted as comparative performance measures rather than absolute diagnostic accuracy [43,44].

## 3. Results

### 3.1. Socio-Demographic, Behavioral, and Environmental Characteristics of Participants

Among the 260 participants enrolled, 131 (50.4%) were recruited from Chemolingot Sub-County Hospital and 129 (49.6%) from Marigat Sub-County Hospital. The study population was nearly equally distributed by sex (51.5% female vs 48.5% male), with a mean age of 27.5 years (IQR: 19–38); min = 54 years, max = 83 years; most participants were aged 15–44 years (63.1%). Agriculture was the predominant occupation (53.5%), and 60.4% reported at least one comorbid condition. Traditional mud-thatched houses were the most common dwelling type (37.3%). Peridomestic and animal-related exposures were widespread: 83.8% slept near animal shelters, 19.2% routinely slept outdoors, 58.9% engaged in outdoor activities after dusk, 63.8% owned dogs, 45.4% slept near dogs, and 83.5% kept other domestic animals. Regular mosquito-net use was reported by 57.3%, as summarized in Table 1.

### 3.2. Bivariate Analysis of Factors Associated with Leishmania Seropositivity

*Leishmania* seropositivity was 18.5% (95% CI: 14.2–23.7) by rK39 RDT and 20.8% (95% CI: 16.2–26.2) by ELISA. In the bivariate analysis, males had higher seropositivity than females (28.6% vs. 13.4%; OR = 2.58, *p* = 0.003). Age was also significantly associated with seropositivity, with 48.8% of children aged < 15 years seropositive vs. 7.5% among participants aged ≥ 45 years (OR = 11.7, *p* < 0.001). Seroprevalence was also higher among participants without formal education (42.5%; *p* < 0.001). Those with at least one comorbid condition (26.1% vs. 12.6%; OR = 2.45, *p* = 0.009). Occupation was not significantly associated with seropositivity (*p* = 0.236), as summarized in Table 1.

Higher seroprevalence was associated with living in mud-thatched houses (*p* < 0.001), residing near animal shelters than those residing near natural habitats alone (23.9% vs. 5.1%; *p* = 0.016) sleeping outdoors (52.0% vs. 13.3%; OR = 7.00, *p* < 0.001), and those owning livestock (23.0% vs. 9.3%; OR = 2.90, *p* = 0.042). Geographical location showed the strongest bivariate association: participants from Tiaty had substantially higher seroprevalence than those from Baringo South (36.6% vs. 4.7%; OR = 11.9, *p* < 0.001). Mosquito-net use was associated with lower odds of seropositivity (8.1% vs. 37.8%; OR = 0.14, *p* < 0.001). Outdoor activities after dusk, dog ownership, and sleeping near dogs were not significantly associated with *Leishmania* seropositivity (*p* > 0.05), as summarized in Table 1.

### 3.3. Multivariable Analysis of Factors Associated with Leishmania Seropositivity

We performed multivariable logistic regression to identify independent predictors of *Leishmania* seropositivity after controlling for potential confounders. We included variables associated with seropositivity at *p* < 0.20 in the bivariate analysis in a forward stepwise model. Adjusted odds ratios (aORs), 95% confidence intervals (CIs), and corresponding *p*-values are presented in Table 2.

After adjustment, sub-County of residence, age group, and dog ownership remained independently associated with *Leishmania* seropositivity. Participants in Tiaty Sub-County had more than 11 times the odds of seropositivity compared with those in Baringo South Sub-County (aOR = 11.30, 95% CI: 1.96–64.97; *p* = 0.007), making geographical location the strongest independent predictor in the final model.

Age group also remained independently associated with seropositivity. Compared with children aged < 15 years, participants aged 15–44 years had 70% lower odds of seropositivity (aOR = 0.30, 95% CI: 0.11–0.93; *p* = 0.037). Participants aged ≥ 45 years also had lower estimated odds than children, although the association was not statistically significant (aOR = 0.25, 95% CI: 0.05–1.25; *p* = 0.091).

Dog ownership was independently associated with seropositivity. Participants without dogs had approximately three times the odds of seropositivity compared with dog owners (aOR = 3.17, 95% CI: 1.06–9.50; *p* = 0.039).

Variables associated with seropositivity in the bivariate analysis, including sex, housing type, mosquito-net use, comorbid illness, livestock ownership, and characteristics of the sleeping environment, were not independently associated with seropositivity after adjustment (all *p* > 0.05). Outdoor activity after dusk was also not retained as an independent predictor. These results suggest that the unadjusted associations may have been influenced by confounding, particularly by geographical location and related contextual factors.

### 3.4. Confirmatory Diagnosis by Demonstration of the Leishmania Parasite

**Figure 2:**
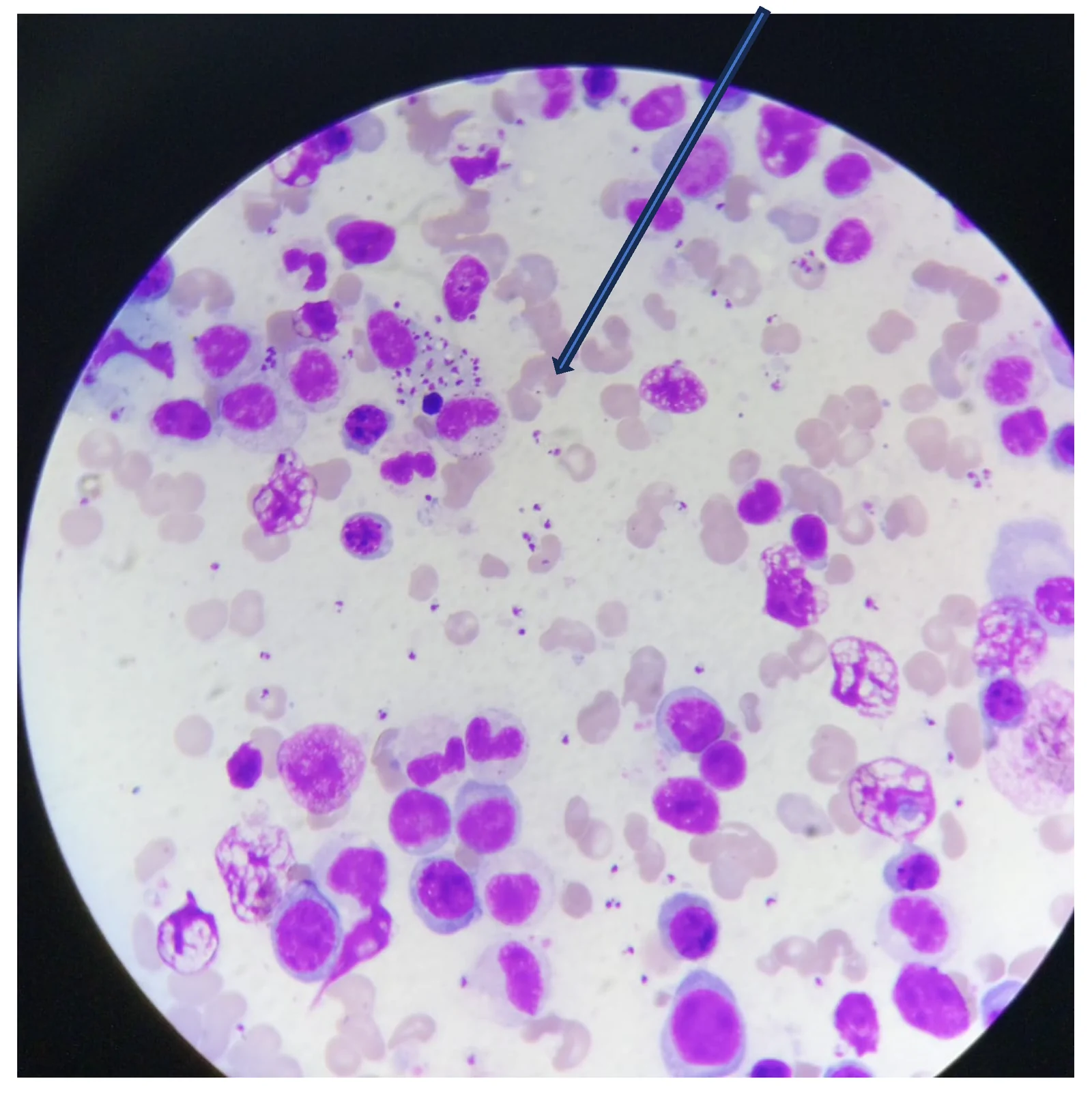
Bone marrow aspirate cytology showing *Leishmania* amastigotes (*Leishmania* bodies) in a case of VL.

Approximately 10 suspected VL cases were confirmed by classical parasitological methods through microscopic detection of *Leishmania* amastigotes (*Leishman–Donovan* bodies), identified by their characteristic pale-blue cytoplasm, red-staining nucleus, and rod-shaped kinetoplast positioned at a right angle to the nucleus (Figure 2).

### 3.5. Model Discrimination and Agreement Between the rK39 RDT and ELISA

The final multivariable logistic regression model demonstrated good discriminatory ability, with an area under the receiver operating characteristic curve (AUC) of 0.87, indicating that it effectively distinguished between ELISA-seropositive and ELISA-seronegative participants.

The rK39 rapid diagnostic test (RDT) and ELISA showed almost perfect agreement (Cohen’s κ = 0.854, *p* < 0.001), with an overall agreement of 95.4% (248/260) (Table 3). ELISA identified 54 of 260 participants (20.8%) as seropositive, whereas the rK39 RDT identified 48 (18.5%). Both assays were positive in 45 participants and negative in 203. Among the 54 ELISA-seropositive participants, 45 (83.3%) tested positive by rK39, while 9 (16.7%) tested negative. Of the 206 ELISA-seronegative participants, 203 (98.5%) tested negative by rK39, whereas 3 (1.5%) tested positive.

Using ELISA as the comparator assay, the rK39 RDT demonstrated a sensitivity of 83.3%, specificity of 98.5%, positive predictive value of 93.8%, and negative predictive value of 95.8%. These findings indicate strong concordance between the assays and the rK39 RDT’s particularly high specificity for detecting anti-*Leishmania* antibodies. However, its comparatively lower sensitivity suggests that rK39 alone may miss some ELISA-seropositive individuals. Therefore, patients with a strong clinical suspicion of visceral leishmaniasis despite a negative rK39 result should undergo further evaluation using ELISA and, where feasible, parasitological or molecular confirmation. Because ELISA is not a definitive reference standard for active visceral leishmaniasis, these estimates reflect comparative rather than absolute diagnostic performance.

### 3.6. Performance of the Prediction Model

The multivariable prediction model correctly classified 221 of 260 participants, for an overall accuracy of 85.0% (Table 4). It correctly identified 30 of the 54 ELISA-seropositive participants (sensitivity, 55.6%) and 191 of the 206 ELISA-seronegative participants (specificity, 92.7%). The model had a positive predictive value of 66.7% and a negative predictive value of 88.8%. The false-positive and false-negative rates were 7.3% and 44.4%, respectively.

The model demonstrated good overall discrimination and high specificity but only moderate sensitivity. It was therefore more effective at identifying ELISA-seronegative participants than at detecting all seropositive participants. The relatively high false-negative rate indicates that the model may miss a substantial proportion of seropositive individuals and should not be used as a stand-alone screening tool. Instead, it may be more useful for risk stratification when combined with clinical assessment and appropriate diagnostic testing.

## 4. Discussion

This study reports a seroprevalence of *Leishmania* infection in humans of 18.5% with the rK39 rapid diagnostic test and 20.8% with ELISA, providing strong evidence of ongoing parasite transmission in Baringo County. The observed seroprevalence was slightly higher than that reported in recent community-based studies from Baringo [20] and several endemic areas of East Africa [27,35,45,46,47,48] and the Middle East [49] and Jordan [50]. However, it remained lower than estimates from hyperendemic regions of Ethiopia [36], Ghana [1] and Chad [51], while being comparable to findings from Bangladesh [52] and Thailand [53]. The observed differences likely reflect variation in study design (hospital-based vs. community-based), transmission patterns, diagnostic methods, populations, ecology, and vector control measures. Despite potential overestimation due to hospital-based sampling, agreement with community surveys indicates sustained, localized VL transmission in Baringo County.

A key finding was the marked geographical disparity in *Leishmania* infection risk between the two study areas. Participants from Tiaty had an adjusted odds ratio of seropositivity more than 11-fold higher than those from Baringo South, confirming Tiaty as the principal transmission hotspot. This elevated risk likely reflects the interaction of vector-favorable ecology, pastoral and outdoor practices, vulnerable housing, and socioeconomic conditions that increase human–sand fly contact and sustain transmission [13]. Its low-lying, semi-arid environment, abundant termite mounds and animal burrows, and extensive *Acacia–Prosopis* vegetation provide favorable breeding and resting habitats for sand flies [20,32]. These ecological conditions interact with pastoral practices including livestock herding, outdoor sleeping, and proximity to animal enclosures to increase human–vector contact. Traditional and temporary dwellings may further enhance exposure by facilitating sand fly entry and providing indoor resting sites. Pastoral mobility, prolonged outdoor activity, proximity to livestock, and limited awareness of vector-borne transmission may collectively sustain leishmaniasis transmission, particularly in Tiaty Sub-county [5,20]. Conversely, the lower seroprevalence observed in Baringo South Sub-County may reflect differences in housing quality, healthcare access, timeliness of diagnosis, community awareness, and local vector ecology [5]. Similar spatial heterogeneity has been documented in other VL-endemic settings in Eastern Africa [45,47,54], Asia [49,55], and Jordan [50], demonstrating the importance of local ecological and socioeconomic conditions in shaping transmission. These findings support prioritizing Tiaty for intensified surveillance, active case detection, prompt diagnosis and treatment, and habitat-based vector control. Geographically targeted interventions that address the area’s specific ecological and social conditions are likely to have a greater impact on public health than uniformly implemented county-wide measures.

Children younger than 15 years were disproportionately affected, with higher age-related risk, particularly among pastoralist communities, which confirms their heightened vulnerability to *Leishmania* infection. These findings align with studies from East Africa [9,25,30,40,50], South Asia [46,49], and the Middle East [51], where children consistently bear the highest VL burden. Their increased susceptibility is likely multifactorial, reflecting immature cell-mediated immunity or absence of preexisting immunity, malnutrition, inadequate personal protection, poor housing, and frequent participation in outdoor activities such as livestock herding, charcoal burning, and beekeeping, which increase exposure to sand fly habitats [20,21,22,54]. In East Africa, intense peri-domestic transmission of *L. donovani* particularly affects children in endemic communities. Conversely, in zoonotic settings such as southern Europe, cumulative exposure may result in higher infection rates among older adults [56]. Although this cross-sectional study could not distinguish behavioral from immunological contributions, both plausibly contributed to the elevated seropositivity among children. These findings support prioritizing children for active surveillance, early diagnosis, and targeted preventive interventions.

Male participants exhibited significantly higher seropositivity than females. This is consistent with reports from multiple endemic regions in East Africa [45,47,57], West Africa [1], Asia [52], and Europe [56]. The observed sex disparity likely reflects the greater occupational and behavioral exposure of male pastoralists, who commonly herd livestock, guard animals overnight, and sleep outdoors during peak sand fly-biting hours, resulting in greater cumulative exposure to infected vectors [20]. Sex-related immunological differences may also contribute to biological susceptibility. Androgens, particularly testosterone, may suppress protective Th1-mediated immunity, whereas estrogens may enhance cellular and humoral immune responses, potentially improving parasite control [58,59]. Transcriptomic and animal studies further suggest that biological sex influences immune responses, disease progression, and drug metabolism [60,61]. However, because most mechanistic evidence is derived from laboratory and animal studies, gender-related exposure patterns provide a more plausible explanation for the observed male predominance. Conversely, studies reporting higher seropositivity among females in some settings [53] may likely reflect differences in local cultural practices, livelihood patterns, and gender roles that influence exposure to sand fly bites. Further human studies are needed to clarify the relative contributions of occupational exposure and biological susceptibility.

Socioeconomic conditions also influence infection risks. Individuals without formal education and those residing in traditional mud-thatched houses experienced significantly higher seroprevalence than their counterparts. These findings align with similar studies in Kenya [20,33,34] and Chad [51]. Limited education may reduce awareness of sand fly ecology, disease transmission, and preventive practices, and delay healthcare-seeking behavior, particularly in pastoralist communities with limited access to health information [5,13]. Likewise, cracked mud walls, earthen floors, and grass-thatched roofs provide suitable resting sites for phlebotomine sand flies and facilitate vector entry into human sleeping areas, thereby increasing indoor transmission. These findings reinforce previous epidemiological findings in East Africa [27,33] and Asia [52,53,62]. Together, these findings demonstrate that education and housing quality are important, modifiable determinants of VL risk and support integrated interventions that combine community health education with housing improvements to reduce human–vector contact.

Peridomestic environmental conditions also contributed to *Leishmania* infection risk. Living near livestock shelters and routinely sleeping outdoors were associated with significantly higher seropositivity, most likely because these conditions increase exposure to nocturnally active sand flies. These findings are consistent with previous studies conducted in Baringo County [33,34]. 

Livestock shelters, animal manure, and accumulated organic waste can provide favorable resting and breeding microhabitats for sand flies. In contrast, domestic animals serve as blood-meal sources that may sustain local vector populations [12,27,32]. Pastoral practices such as sleeping outdoors, nighttime livestock guarding, and resting beneath Acacia trees during hot weather may further increase exposure to sand flies during peak activity. Comparable associations have been reported in Kenya and Ethiopia [33,47,48]. High daytime temperatures may reinforce these exposure patterns by encouraging nighttime herding and outdoor sleeping [20]. These environmental and behavioral factors may act synergistically to sustain transmission in pastoral communities, highlighting the need for improved livestock housing, effective management of manure and organic waste, reduced outdoor exposure, and targeted peri-domestic vector control.

Although non-use of mosquito nets was associated with higher seropositivity in the unadjusted analysis, evidence regarding their effectiveness against VL remains inconsistent across endemic settings in Eastern Africa [27] and Chad [51]. The limited protection observed in Baringo may be partly explained by the predominantly outdoor-biting behavior of *Phlebotomus martini*, which reduces the effectiveness of conventional insecticide-treated nets when individuals are exposed before retiring indoors or while sleeping outside [20]. Bed nets should therefore be promoted as one component of an integrated vector-management strategy rather than as a stand-alone intervention. Complementary measures should include peri-domestic environmental management, targeted residual insecticide spraying, housing improvement, personal protection during outdoor activities, and context-specific community education.

Participants with underlying comorbid illnesses also exhibited higher *Leishmania* seropositivity. This aligns with earlier observations from Bangladesh [52]. Concurrent infections, malnutrition, and chronic illnesses may impair host immune responses, increasing susceptibility to infection and progression to clinical disease [20,27,35]. Given the high burden of malaria, helminth infections, and undernutrition in endemic regions of Kenya, integrating routine screening and management of comorbid conditions into VL surveillance and clinical care could facilitate earlier diagnosis and improve patient outcomes.

After multivariable adjustment, household dog ownership was not independently associated with human Leishmania seropositivity. This finding is biologically plausible because VL in East Africa is caused predominantly by *L. donovani*, with transmission largely anthroponotic and humans as the principal reservoir, unlike zoonotic L. infantum transmission, in which domestic dogs play a central epidemiological role [12,27]. Similar observations have been reported in Jordan [50], India [55], Brazil [7], and Ethiopia [47]. However, studies elsewhere in Chad [51], West Pokot, Kenya [11], Thailand [53], Iran [49], and Spain [56] demonstrated that close contact with dogs was associated with increased infection risk. Dog ownership in Baringo may therefore represent a proxy for broader pastoral and environmental conditions rather than an independent risk factor for human infection. Nevertheless, domestic animals may still contribute indirectly by sustaining sand fly populations and increasing vector abundance around households. Future One Health investigations incorporating molecular parasite typing, reservoir studies, and entomological surveillance are required to clarify the contribution of domestic animals to local transmission dynamics.

Several established risk factors were associated with *Leishmania* seropositivity in univariable analyses but lost significance after adjustment, suggesting confounding by geographical, ecological, and socioeconomic conditions. These household and behavioral exposures may therefore reflect residence in high-transmission settings rather than independently predict infection. Similar findings across Eastern Africa emphasize the need to account for contextual confounding and spatial heterogeneity in VL transmission. Control strategies should therefore target high-risk areas and their underlying ecological and socioeconomic conditions rather than focus solely on individual household factors.

The multivariable prediction model demonstrated good discriminatory performance (AUC = 0.87), indicating that demographic, environmental, behavioral, and socioeconomic variables effectively identified individuals at increased risk of *Leishmania* infection. The remaining unexplained variation likely reflects unmeasured determinants, including local vector density, individual exposure intensity, micro-environmental variation, and parasite circulation within animal reservoirs. This performance is comparable to that reported in other East African studies and supports the model’s utility for risk stratification and targeted surveillance in endemic settings [20]. Future studies integrating geospatial analyses, entomological surveillance, and molecular epidemiology may further improve predictive accuracy and refine risk mapping in endemic areas.

Diagnostic agreement between the rK39 rapid diagnostic test and ELISA was excellent, with near-perfect concordance (κ = 0.854), high specificity (98.5%), and positive predictive value (93.8%), supporting the continued use of rK39 as a frontline diagnostic tool in resource-limited settings. However, its lower sensitivity indicates that reliance on rK39 alone may underestimate early or asymptomatic infections, consistent with previous reports from East Africa [27]. Sequential diagnostic algorithms that combine rapid screening with ELISA or molecular confirmation in suspected false-negative cases could improve surveillance while remaining clinically feasible.

### Limitations

This study had several limitations. The hospital-based cross-sectional design may have introduced selection bias because patients attending health facilities may not represent the wider community, particularly where socioeconomic circumstances influence access to healthcare. Recruitment may also have disproportionately captured severe cases, potentially conflating factors associated with disease severity—such as malnutrition—with predictors of infection. Consequently, seroprevalence may have been overestimated, generalizability is limited, and causal relationships cannot be established. Reliance on rK39 and ELISA demonstrated serological agreement rather than definitive diagnostic accuracy and could not distinguish active from past infection. Diagnostic misclassification may also have resulted from clinically similar endemic diseases, single-sample testing, and unassessed variability in rK39 interpretation. Furthermore, self-reported exposures and unmeasured environmental factors may have introduced information bias and residual confounding. Molecular confirmation is therefore needed to identify the circulating Leishmania species. Despite these limitations, the study provides valuable evidence of ongoing transmission and identifies factors relevant to targeted surveillance and integrated One Health interventions.

## 5. Conclusions

Overall, this study demonstrates that *Leishmania* seroprevalence among patients with suspected VL in Baringo County was substantial and geographically heterogeneous, with residence in Tiaty sub-County and younger age emerging as key predictors. Transmission reflects interacting demographic, behavioral, socioeconomic, housing, and ecological factors that increase human–vector contact. Control efforts should prioritize Tiaty through child-focused surveillance, active case detection, prompt treatment, integrated vector and habitat management, housing improvements, personal protection, and culturally appropriate education. A targeted One Health approach integrating human, animal, and entomological surveillance may be more effective than uniform countywide interventions. It can support WHO in advancing regional and national goals toward eliminating Neglected Tropical Diseases in Kenya and other endemic regions of East Africa.

## Figures and Tables

**Table 1:** Demographic characteristics of the study participants and bivariate Association between selected risk factors and *Leishmania* seropositivity in Baringo County, Kenya (N = 260).

Variable	Categories	Frequency (%)	Negative	Positive	Fishers Test
Sex	Female	134 (51.5)	116	18	0.003
	Male	126 (48.5)	90	36	
Age group	Children (<15 years)	43 (16.5)	22	21	<0.001
	Youth and young adults 15–44 years	164 (63.1)	135	29	
	Older adults > 45	53 (20.4)	49	4	
Education attainment	None	87 (33.5)	50	37	<0.001
	Primary	84 (32.3)	77	7	
	Secondary or higher	89 (34.2)	79	10	
Economic activity	Nonagricultural	121 (46.5)	92	29	0.236
	Agricultural	139 (53.5)	114	25	
Other illness	No	103 (39.6)	90	13	0.009
	Yes	157 (60.4)	116	41	
Type of housing	Hut	97 (37.3)	57	40	<0.001
	Mabati	95 (36.5)	87	8	
	Permanent	68 (26.2)	62	6	
Ecological habitat	None	3 (1.2)	3	0	0.016
	Natural (termite/vegetation)	39 (15.0)	37	2	
	Peridomestic (animal shed)	218 (83.8)	166	52	
Sleeping outdoors	No	210 (80.8)	182	28	<0.001
	Yes	50 (19.2)	24	26	
Subcounty of residence	Baringo South (Marigat Hospital)	129 (49.6)	123	6	<0.001
	Tiaty (Chemolingot Hospital)	131 (50.4)	83	48	
Outdoor activities after dusk	No	107 (41.2)	90	17	0.105
	Yes	153 (58.8)	116	37	
Use of mosquito nets	No	111 (42.7)	69	42	<0.001
	Yes	149 (57.3)	137	12	
Dog ownership	No	94 (36.2)	79	15	0.150
	Yes	166 (63.8)	127	39	
Sleep near dogs	No	48 (28.9)	36	12	0.444
	Yes	118 (71.1)	91	27	
Presence of other domestic animals	No	43 (16.5)	39	4	0.042
	Yes	217 (83.5)	167	50	

**Table 2:** Multivariate logistic model analysis of socio-demographic, behavioral, and environmental factors associated with human leishmaniasis in Baringo County, Kenya.

Variable	Covariable	Reference Category	OR (95% CI)	*p*-Value
Sex	Female	Male	0.5 (0.2–1.19)	0.116
Age	15–44 yrs 45 yrs	Children (<15 years)	0.3 (0.11–0.93) 0.25 (0.05–1.25)	0.037 0.91
Education	Primary Secondary or higher	None	0.38 (0.11–1.24) 0.74 (0.22–2.53)	1.109 0.633
Subcounty	Tiaty	Baringo south	11.3 (1.96–64.9)	0.007
Type of house	Mabati permanent	Hut	0.79 (0.21–2.95) 0.45 (0.10–1.99)	0.723 0.296
Dawn/dusk activities	No	Yes	0.89 (0.35–2.25)	0.807
Other illness	No	Yes	0.84 (0.33–2.08)	0.703
Dog ownership	No	Yes	3.17 (1.06–9.5)	0.039
Use of nets	no	Yes	1.09 (0.32–3.71)	0.887
Other animals	no	Yes	4.84 (0.79–29.4)	0.087
Ecology habitat	Non-peridomestic	peridomestic	0.55 (0.081–3.7)	0.537

**Table 3:** Agreement between the rK39 rapid diagnostic test (RDT) and ELISA.

	ELISA Positive	ELISA Negative	Total
RDT Positive	True Positive (45; 93.8%)	False Positive (3; 6.2%)	48
RDT Negative	False Negative (9; 4.2%)	True Negative (203; 95.8%)	212
Total	54	206	260

**Table 4:** Multivariable prediction model.

Performance	Value	Interpretation
Sensitivity	55.6%	Moderate ability to identify ELISA-positive participants
Specificity	92.7%	Excellent ability to identify ELISA-negative participants
Positive Predictive Value	66.7%	Two-thirds of predicted positives were truly positive
Negative Predictive Value	88.8%	Most predicted negatives were truly negative
Overall Accuracy	85.0%	Good overall classification performance
False-positive rate	7.3%	Participants that were incorrectly classified as positive
False-negative rate	44.4%	Proportion of infected participants that were missed

## Data Availability

The original contributions presented in this study are included in the article. Further inquiries can be directed to the corresponding author.

## Abbreviations

VL	Visceral Leishmaniasis
WHO	World Health Organization
PKDL	Post-kala-azar dermal leishmaniasis
NTD	Neglected Tropical Diseases
RDT	Rapid diagnostic Test
ELISA	Enzyme-linked immunosorbent assay
KEMRI	Kenya Medical Research Institute

## References

[1] Akuffo R., Wilson M., Sarfo B., Attram N., Mosore M.-T., Yeboah C., Cruz I., Ruiz-Postigo J.-A., Boakye D., Moreno J. (2021). Prevalence of *Leishmania* infection in three communities of Oti Region, Ghana. PLoS Neglected Trop. Dis..

[2] Tedla D.G., Bariagabr F.H., Abreha H.H. (2018). Incidence and Trends of Leishmaniasis and Its Risk Factors in Humera, Western Tigray. J. Parasitol. Res..

[3] Andreottola V., Santucci C., Bellini T., Matarese S., Canzoneri F., Dell’orso G., Finetti M., Fioredda F., Mesini A., Piccotti E. (2025). Visceral Leishmaniasis in Pediatrics: A Case Series and a Narrative Review with Global Insights. Trop. Med. Infect. Dis..

[4] Okindo E.G., Kasili S., Kutima H.L., Mutai J.M. (2016). Socioeconomic Impacts of Leishmaniases on Households of Marigat Sub County, Baringo County of Kenya. J. Trop. Dis..

[5] Maingi H.N., Ndichu M., Chege J.N., Karanja D.N., Sentamu D.N., Lokonon B.E., Matoke-Muhia D., Ngowi H., Bonfoh B. (2026). Community Drivers of *Leishmania* Infection, Transmission and Control in Baringo County, Kenya: Implications for Integrated One Health Intervention Strategies. Trop. Med. Infect. Dis..

[6] Bouattour A., Amri A., Belkhiria J.A., Rhim A., Fezaa O., Gantier J.-C., M’ghirbi Y. (2021). Canine leishmaniosis in Tunisia: Growing prevalence, larger zones of infection. PLoS Neglected Trop. Dis..

[7] Ponte C.B., Souza N.C., Cavalcante M.N., Barral A.M.P., de Aquino D.M.C., Caldas A.d.J.M. (2011). Risk factors for *Leishmania chagasi* infection in an endemic area in Raposa, State of Maranhão, Brazil. Rev. Soc. Bras. Med. Trop..

[8] Ullah W., Yen T.-Y., Niaz S., Nasreen N., Tsai Y.-F., Rodriguez-Vivas R.I., Khan A., Tsai K.-H. (2023). Distribution and Risk of Cutaneous Leishmaniasis in Khyber Pakhtunkhwa, Pakistan. Trop. Med. Infect. Dis..

[9] Abass E., Kang C., Martinkovic F., Semião-Santos S.J., Sundar S., Walden P., Piarroux R., El Harith A., Lohoff M., Steinhoff U. (2015). Heterogeneity of Leishmania donovani Parasites Complicates Diagnosis of Visceral Leishmaniasis: Comparison of Different Serological Tests in Three Endemic Regions. PLoS ONE.

[10] Kanyina E.W. (2020). Characterization of visceral leishmaniasis outbreak, Marsabit County, Kenya, 2014. BMC Public Health.

[11] Abdullahi B., Mutiso J., Gicheru M. (2024). Social Demographic Characteristics Associated with Visceral Leishmaniasis in West Pokot, Kenya. Am. J. Trop. Med. Hyg..

[12] Gebremedhin E.Z., Weya W., Dima C., Zewde D., Presti V.D.M.L., Vitale M. (2022). High seroprevalence of *Leishmania infantum* infection in dogs and its associated risk factors in selected towns of Southwest and West Shewa zones of Oromia, Ethiopia. Veter-Med. Sci..

[13] O’BRien K., Nyakundi H., Kitondo M., Olawepo J., McCreedy K., Tieskens K., Wamai R. (2025). Mapping visceral leishmaniasis and examining environmental influences in Baringo County, Kenya. Acta Trop..

[14] Wiggins T.J., Peng R., Bushnell R.V., Tobin J.K., MacLeod D.A., Du K., Tobin G.J., Dollery S.J. (2024). Instrument-Free Point-of-Care Diagnostic for Leishmania Parasites. Diagnostics.

[15] Grifferty G., Shirley H., O’bRien K., Hirsch J.L., Orriols A.M., Amechi K.L., Lo J., Chanda N., El Hamzaoui S., Kahn J. (2023). The leishmaniases in Kenya: A scoping review. PLoS Neglected Trop. Dis..

[16] Tadese D., Hailu A., Bekele F., Belay S. (2019). An epidemiological study of visceral leishmaniasis in North East Ethiopia using serological and leishmanin skin tests. PLoS ONE.

[17] Kennedy G.C., O’bRien K., Nyakundi H., Kitondo M., Biwott W., Wamai R.G. (2024). Visceral leishmaniasis follow-up and treatment outcomes in Tiaty East and West sub-counties, Kenya: Cure, relapse, and Post Kala-azar Dermal Leishmaniasis. PLoS ONE.

[18] Abdullahi B., Mutiso J., Maloba F., Macharia J., Riongoita M., Gicheru M. (2022). Climate Change and Environmental Influence on Prevalence of Visceral Leishmaniasis in West Pokot County, Kenya. J. Trop. Med..

[19] O’bRien K., Kennedy G., Nyakundi H., Kitondo M., Pembee V., Biwott W., Wamai R. (2024). Environmental Risk Factors for Visceral Leishmaniasis: An Analysis of Housing Types and Behavioral Factors in Baringo County, Kenya. Am. J. Trop. Med. Hyg..

[20] Yator C., Chebii K. (2025). Risk Factors of Visceral Leishmaniasis among Residents of Chemolingot, Baringo Kenya. Afro-Egypt J. Infect. Endem. Dis..

[21] Prestes-Carneiro L.E., Spir P.R.N., Fontanesi M., Pereira Garcia K.G., Silva F.A.D., Flores E.F., Vasconcelos D.D.M. (2019). Unusual manifestations of visceral leishmaniasis in children: A case series and its spatial dispersion in the western region of São Paulo state, Brazil. BMC Infect. Dis..

[22] Dondi A., Manieri E., Gambuti G., Varani S., Campoli C., Zama D., Pierantoni L., Baldazzi M., Prete A., Attard L. (2024). A 10-Year Retrospective Study on Pediatric Visceral Leishmaniasis in a European Endemic Area: Diagnostic and Short-Course Therapeutic Strategies. Healthcare.

[23] Roberts T., Keddie S.H., Rattanavong S., Gomez S.R., Bradley J., Keogh R.H., Bärenbold O., Falconer J., Mens P.F., Hopkins H. (2023). Accuracy of the direct agglutination test for diagnosis of visceral leishmaniasis: A systematic review and meta-analysis. BMC Infect. Dis..

[24] El Harith A., Osman H.A., Mansour D., Semiao-Santos S.J., Abass E. (2025). Direct agglutination test: Evolution and significance as a simple and safe alternative to tissue aspiration procedures in the diagnosis of visceral leishmaniasis. PLoS ONE.

[25] Chandra H., Chandra S., Kaushik R.M. (2013). Visceral Leishmaniasis with Associated Common, Uncommon, and Atypical Morphological Features on Bone Marrow Aspirate Cytology in Nonendemic Region. J. Trop. Med..

[26] Manomat J., Leelayoova S., Bualert L., Tan-Ariya P., Siripattanapipong S., Mungthin M., Naaglor T., Piyaraj P. (2017). Prevalence and risk factors associated with *Leishmania* infection in Trang Province, southern Thailand. PLoS Negl. Trop. Dis..

[27] Ketema H., Weldegebreal F., Gemechu A., Gobena T. (2022). Seroprevalence of visceral leishmaniasis and its associated factors among asymptomatic pastoral community of Dire District, Borena zone, Oromia Region, Ethiopia. Front. Public Health.

[28] Hamarsheh O., Nasereddin A., Damaj S., Sawalha S., Al-Jawabreh H., Azmi K., Amro A., Ereqat S., Abdeen Z., Al-Jawabreh A. (2012). Serological and molecular survey of *Leishmania* parasites in apparently healthy dogs in the West Bank, Palestine. Parasites Vectors.

[29] Mahdavi R., Shams-Eldin H., Witt S., Latz A., Heinz D., Fresco-Taboada A., Aira C., Hübner M.P., Sukyte D., Visekruna A. (2023). Development of a Novel Enzyme-Linked Immunosorbent Assay and Lateral Flow Test System for Improved Serodiagnosis of Visceral Leishmaniasis in Different Areas of Endemicity. Microbiol. Spectr..

[30] van Dijk N.J., Hagos D.G., Huggins D.M., Carrillo E., Ajala S., Chicharro C., Kiptanui D., Solana J.C., Abner E., Wolday D. (2024). Simplified molecular diagnosis of visceral leishmaniasis: Laboratory evaluation of miniature direct-on-blood PCR nucleic acid lateral flow immunoassay. PLoS Negl. Trop. Dis..

[31] Ryan J.R., Mbui J., Rashid J.R., Wasunna M.K., Kirigi G., Magiri C., Kinoti D., Ngumbi P.M., Martin S.K., Odera S.O. (2006). Spatial clustering and epidemiological aspects of visceral leishmaniasis in two endemic villages, baringo district, kenya. Am. J. Trop. Med. Hyg..

[32] Hassaballa I.B., Sole C.L., Cheseto X., Torto B., Tchouassi D.P. (2021). Afrotropical sand fly-host plant relationships in a leishmaniasis endemic area, Kenya. PLoS Negl. Trop. Dis..

[33] Kiptui E.G., Kiprono S.J., Mengich G.J. (2021). Risk factors of visceral leishmaniasis among residents of Baringo County, Kenya. Int. J. Community Med. Public Health.

[34] Gwandi M.P., Odongo A.O., Kirira P.G., Jeruto E. (2022). Risk Factors Associated with Leishmaniasis among Residents of Rural Marigat Sub-County, Baringo County—Kenya. Int. J. Trop. Dis. Health.

[35] Alebie G., Worku A., Yohannes S., Urga B., Hailu A., Tadesse D. (2019). Epidemiology of visceral leishmaniasis in Shebelle Zone of Somali Region, eastern Ethiopia. Parasites Vectors.

[36] Gize A., Workineh A., Hailu T. (2020). A trend prevalence of visceral Leishmaniasis in West Armachiho District, Amhara Region, Northwest Ethiopia. Trop. Dis. Travel Med. Vaccines.

[37] Abass E. (2020). Leishmania donovani infection in Eastern Sudan: Comparing direct agglutination and rK39 rapid test for diagnosis-a retrospective study. Asian Pac. J. Trop. Med..

[38] Reiczigel J., Földi J., Ózsvári L. (2010). Exact confidence limits for prevalence of a disease with an imperfect diagnostic test. Epidemiol. Infect..

[39] Hazra A., Gogtay N. (2016). Biostatistics series module 3: Comparing groups: Numerical variables. Indian J. Dermatol..

[40] Hazra A., Gogtay N. (2016). Biostatistics series module 6: Correlation and linear regression. Indian J. Dermatol..

[41] Zhang Z. (2016). Model building strategy for logistic regression: Purposeful selection. Ann. Transl. Med..

[42] Ailobhio D.T., Ikughur J.A. (2024). A Review of Some Goodness-of-Fit Tests for Logistic Regression Model. Asian J. Probab. Stat..

[43] Bangdiwala S.I., Shankar V. (2013). The agreement chart. BMC Med. Res. Methodol..

[44] Dhamnetiya D., Jha R.P., Shalini S., Bhattacharyya K. (2022). How to Analyze the Diagnostic Performance of a New Test? Explained with Illustrations. J. Lab. Physicians.

[45] Odoch W.D., Olobo J.O. (2013). Prevalence of kala-azar infection in Pokot county, Amudat district, Northeastern Uganda. Open J. Epidemiol..

[46] Ayehu A., Aschale Y., Lemma W., Alebel A., Worku L., Jejaw A., Bayih A.G. (2018). Seroprevalence of Asymptomatic *Leishmania donovani* among Laborers and Associated Risk Factors in Agricultural Camps of West Armachiho District, Northwest Ethiopia: A Cross-Sectional Study. J. Parasitol. Res..

[47] Bsrat A., Berhe M., Gadissa E., Taddele H., Tekle Y., Hagos Y., Abera A., G/Micael M., Alemayhu T., Gugsa G. (2018). Serological investigation of visceral *Leishmania* infection in human and its associated risk factors in Welkait District, Western Tigray, Ethiopia. Parasite Epidemiol. Control.

[48] Ismail A., Yared S., Dugassa S., Abera A., Animut A., Erko B., Gebresilassie A. (2023). Seroprevalence of visceral leishmaniasis and its associated factors among asymptomatic individuals visiting Denan health center, southeastern Ethiopia. Trop. Dis. Travel Med. Vaccines.

[49] Gigloo A.L., Sarkari B., Rezaei Z., Hatam G.R., Davami M.H. (2018). Asymptomatic *Leishmania* Infected Children: A Seroprevalence and Molecular Survey in a Rural Area of Fars Province, Southern Iran. J. Trop. Med..

[50] Obaidat M.M., Roess A. (2019). Nationwide seroprevalence, spatial distribution and risk factors of *Leishmania* in Jordan. Asian Pac. J. Trop. Biomed..

[51] Yiyikbo A.Y., Dikti J.V., Kodindo I.D., Kamaya B., Souhoudi F., Paggo B.O.R., Bonaventure F., Suitombaye N.Y., Djibagaou A.D., Akouya A. (2025). High seroprevalence and associated risk factors of visceral leishmaniasis in the Léré Health District, Chad. Sahel. J. Responsible One Health.

[52] Bhowmick A.R.B., Khanum H. (2017). Prevalence of visceral leishmaniasis, risk factors and associated disorders: Knowledge of inhabitants and professionals in Fulbaria, Mymensingh. Bangladesh J. Zool..

[53] Sriwongpan P., Nedsuwan S., Manomat J., Charoensakulchai S., Lacharojana K., Sankwan J., Kobpungton N., Sriwongpun T., Leelayoova S., Mungthin M. (2021). Prevalence and associated risk factors of *Leishmania* infection among immunocompetent hosts, a community-based study in Chiang Rai, Thailand. PLoS Neglected Trop. Dis..

[54] Salih O.A.M.M., Nail A.M., Modawe G.A., Swar M.O., Ahmed M.H., Khalil A., Satti A.B., Abuzeid N. (2020). Risk factors of inpatients mortality of visceral leishmaniasis, Khartoum State, Sudan. J. Glob. Infect. Dis..

[55] Singh N.S. (2019). A Review on Major Risk Factors and Current Status of Visceral Leishmaniasis in North India. Am. J. Èntomol..

[56] Ibarra-Meneses A.V., Carrillo E., Nieto J., Sánchez C., Ortega S., Estirado A., Zamalloa P.L., Sanz J.C., García-Comas L., Ordobás M. (2019). Prevalence of asymptomatic *Leishmania* infection and associated risk factors, after an outbreak in the south-western Madrid region, Spain, 2015. Eurosurveillance.

[57] Geto A.K., Berihun G., Berhanu L., Desye B., Daba C. (2024). Prevalence of human visceral leishmaniasis and its risk factors in Eastern Africa: A systematic review and meta-analysis. Front. Public Health.

[58] Battistoni O., Huston R.H., Verma C., Pacheco-Fernandez T., Abul-Khoudoud S., Campbell A., Satoskar A.R. (2025). Understanding Sex-biases in Kinetoplastid Infections: Leishmaniasis and Trypanosomiasis. Expert Rev. Mol. Med..

[59] Pandey K., Singh D., Bimal S., Murti K., Das P. (2014). Association of testosterone and cholesterol level in modulation of immunity and severity of disease in visceral leishmaniasis patients—A preliminary study. Am. J. Immunol..

[60] Lockard R.D., Wilson M.E., Rodríguez N.E. (2019). Sex-Related Differences in Immune Response and Symptomatic Manifestations to Infection with *Leishmania* Species. J. Immunol. Res..

[61] Honecker B., Hansen C.S., Lotter H. (2026). Sex in Immune Cells and Parasitic Diseases—A Complex Relationship. Immunol. Rev..

[62] Joshi A.B., Banjara M.R., Das M.L., Ghale P., Pant K.R., Pyakurel U.R., Dahal G., Paudel K.P., Das C.L., Kroeger A. (2024). Epidemiological, Serological, and Entomological Investigation of New Visceral Leishmaniasis Foci in Nepal. Am. J. Trop. Med. Hyg..

